# Topics of Public Interest

**Published:** 1913-05

**Authors:** 


### TOPICS OF PUBLIC INTEREST.

Beri Beri and Unpolished Rice. Odake and Kozai claim to have solved the puzzle as to the connection between unpolished rice and beri beri. From the seed coats not only of rice but of wheat, barley, oats, etc., they have isolated an alkaloid which they call orizamine, and which they claim to be essential to health, along with the proteid and other nutritive constituents of the seeds.

Liability of Druggists. The Court of Appeals at Albany, in the case of Fanny E. Wilson of Brockport against a Buffalo druggist, has ruled that a druggist, dispensing a prepared compound, under his own label, is liable for any damages resulting from its use, in spite of the guarantee as to composition and effects given by the manufacturer. In the present instance, a blood remedy, stated to contain only vegetable ingredients, produced the well known effect of mercury in loosening the teeth. We can't say that our sympathies are either with the druggist, the patient or the manufacturer.

To Amend the Public Health Law, Relative to Medical Licenses.—Introduced by Mr. Seeley, read twice and ordered printed, and when printed to be committed to the Committee on Public Health, reported favorably from said committee with amendments, and ordered reprinted as amended and when reprinted to be referred to the Commitee of the Whole.

Section 1.—Section one hundred and sixty of chapter forty-nine of the laws of nineteen hundred and nine, entitled "An act in relation to the public health, constituting chapter forty-five of the consolidated laws," is hereby amended by adding thereto a new subdivision to be known as subdivision nine, and to read as follows:

9. *"Unprofessional conduct" means and shall include the following acts or conduct by or on the part of a practitioner of medicine*:

(a) *Advertising either in his own name or in the name of another person, firm, association of corporation, in any news-*

*paper, pamphlet, or other printed paper or document, or* [by] *writing letters or causing them to be written, or employing capper, solicitor or drummer to secure patients, wherein or whereby the* MEDICAL PRACTITIONER HOLDS HIMSELF OR HERSELF OUT TO CURE DISEASES OR DEFECTS OF THE SEXUAL ORGANS, OR TO CURE CHRONIC OR INCURABLE *diseases, or for being employed by any person, firm, association or corporation so advertising or announcing.*

(b) ANNOUNCING PROFESSIONAL SERVICE WITHOUT COMPENSATION OR *the acceptance of fees in consideration of the assurance that an incurable disease may be permanently cured.*

(c) *Wilfully betraying a professional secret.*

(d) *Habitual drunkenness or addiction to drugs.*

(e) *Dividing or promising to divide a fee with another physician, or accepting a divided fee, without the knowledge of the patient or the person paying such fee.*

[The employment of any capper, solicitor, or drummer for securing patients, or the division of fees or promise of division of fees or the payment of money to any person or persons or of any other valuable thing in return for service in securing patients.]

(f) ANY OTHER ACTION NOT CONSONANT WITH GOOD MORALS, OR ANYTHING DONE OR SAID DETRIMENTAL TO THE HEALTH OR MORALS OF OTHERS.

* * * * * * The regents may revoke the license of a practitioner of medicine, or annul his registration, or do both, *or suspend a practitioner of medicine from the practice of his profession for any length of time,* in any of the following cases: * * *

(c) *Who is guilty of unprofessional conduct, as defined in this article; or* * * *

This bill is opposed by the Western N. Y. Medical Society, President, Dr. R. A. Toms of Kenmore; Secretary, Dr. E. L. Downey, Middleport. While its general objects are laudable, it is also a general principle that it is unwise to attempt to make men gentlemen by law and that, in so doing, grave injustice is liable to occur to men of good intentions and to those committing very minor indiscretions, by putting punitive powers in the hands of men not trained or authorized to perform judiciary functions. And, as worded, the bill might be interpreted so as to interfere with perfectly legitimate methods of publicity and distribution of information, such as contributing to periodicals, sending reprints, use of signs, etc.

---

EXPLANATION—Matter in italics is new; matter in brackets [ ] is old to be omitted.

Sheppard and Enoch Pratt Hospital, of Towson, Md., Edward N. Brush, M. D., Superintendent, will celebrate its 60th anniversary just before or after the Congress of American Physicians at Washington, in May.

---

The Babies Milk Dispensary of Buffalo, 1911, dispensed 22,000 quarts at a cost of $6,500; in 1912, 42,000 quarts at a cost of $10,000. It is hoped that the growth of the work will further diminish the cost per quart, which, as may be noted, was nearly thirty cents for 1911 and a little less than twenty-five cents in 1912. Support is urgently needed. Contributions may be sent to Miss Minerva Newhall, Secretary, 241 Swan street.

---

The Hospital Aid Association of Lockport has raised $3000 toward a new building.

---

"All Poisons. All of our so-called curative agents (drugs) are poisons and, as a consequence, every one diminishes the vitality of those who take them."—Prof. Alonzo Clark, M. D., New York College of Physicians and Surgeons.

This is certainly true of most alkaloids and glucosides, and of many metals and metaloids, commonly classed as alteratives. But, is it true of drugs logically used to restore an equilibrium between acidity and alkalinity, of mineral oil and agar-agar and other substances acting mechanically on the bowel, of ferments properly used to supply deficiencies, of iron, iodothyrine, and other extracts of secreting organs? And, in the practical sense, is it true of cathartics, internal antiseptics, and various drugs employed to restore normal tone to the heart and vessels, of strychnine when reflexes do not follow stimuli as they normally should and of a host of other drugs, rationally used? Disease consists largely of chemic abnormalities. It is inevitable that it must be combated by chemical agents, in other words—drugs.

---

Open Air Schools. New York has fourteen, with a capacity for 626 children. All other states have forty-one, with a capacity for 1229. Just how the "capacity" of an open air school is so closely limited, we do not understand.

---

Tall Man. Julius Laubach of Brussels, 7 feet 2 inches high, has come to Chicago for an operation on his legs, which are bowed, following a fall while ringing a bell in the cathedral at Cologne. He estimates that his normal height should be 7 feet 6 inches.

An Adult Parthenogenetic Female Frog. On February 28, a reception was given by the Manhattan Medical Society, New York, to Dr. Jacques Loeb, of the Rockfeller Institute for Medical Research, at which he spoke on "Some Recent Experiments in Artificial Parthenogenesis." He is reported to have stated that a female frog, thus produced from an artificially fertilized egg, had reached adult life and, on examination after accidental death, had been found to have normally developed organs throughout, including ovaries containing apparently normal ova.—*Boston M. and S. Jour.*

---

A Heavy Woman. Melissa Cooper, a negress, who died on March 15, at Grayson, Gwinnett County, Ga., is said to have weighed 613 pounds at the time of her death. Presumably some pituitary lesion accounted for this phenomenon.—*Boston M. and S. Jour.*

---

Graduate Nurse Wanted as Hospital Superintendent in Madura, South India. Strong constitution, good education, good hospital record, executive ability and deep spiritual life are the requirements. The salary is $500 a year and quarters, but not board, which costs about $13 a month. Permanent appointees receive $250 as outfit allowance and traveling expenses, allowance for language teacher and vacation expenses. A vacation of about a month is allowed each year and a furlough of one year in seven. The hospital has 50 beds, and about 12,000 resident and out-patients are treated annually. Inquire of Mr. Wilbert B. Smith, 600 Lexington avenue, New York

---

Small Pox. Eight cases have developed among the employes of a railroad shop in Elmira.

---

Death of Quadruplet. The *Boston M. and S. Jour.,* August 15, 1912, noted the birth of quadruplets in Dorchester, Mass. All four survived till March 21, 1913, when the first born, a girl, died of broncho-pneumonia. This is regarded as the only recorded instance of the survival of quadruplets for any considerable period.

---

Opposition to Niagara County Tuberculosis Hospital. Dr. John A. Rafter, Mayor of N. Tonawanda, is opposing the efforts of the State Charities Aid Association, on the ground that the climate and low altitude of Niagara County are not adapted to the treatment of this disease. The control of the tuberculosis problem has, of late years, gotten somewhat out of the hands of the medical profession and there is a possibility of advancing the spark of enthusiasm beyond the supply of accurate knowledge so as to cause knocking. We have personally seen the popular and, to some degree, professional notion regarding the climatic treatment of tuberculosis, box the compass from the warmth, moisture and low altitude of Florida, to the warmth, dryness and fairly high altitude of the western plateau, and then to the cold, more or less humid and high altitude of the mountains of New York, North Carolina, etc. We have also seen cases recover in their original environment. Hence, we have no disposition to take sides in this dispute. But Dr. Rafter has done a great service in raising a strictly medical issue just at the time when the campaign for county and other local tuberculosis hospitals has begun to be successful and is spreading over the country by the contagion of example. Hitherto, the obstacle interposed by officials has been economic, either in the narrow sense of hesitating at an expense, or in the broader sense of discouraging public assistance; or the economic problem of the relative merits and expense of local and of state care has been considered, or there has been a dispute as to the relative merits of different sites. While Dr. Rafter's attitude will be a disappointment to many of the lay workers in this cause, and is opposed to individual professional opinions, the question which he raises is of too great importance and too far reaching to be ignored. It should be thoroughly discussed and settled as definitely as possible by present information, before a universal system of local care is instituted. The delay will cause some individual sacrifice but, in the long run, it is worth while to be very sure that we are right before we go ahead any farther.

---

COMPULSORY VACCINATION has been rigorously carried out at Niagara Falls, Ont., on account of a threat of quarantine, if necessary inforced by troops, from the Provincial Board of Health. Dr. Wilson and his associates on the municipal health board resigned on account of lack of co-operation by the aldermen, and Dr. Harris, Harry Hobson, C. V. Bradford and E. Misener, were appointed on the new board. On account of the secretion of some cases of smallpox, a house to house inspection has been made. A fair building has been used as a quarantine hospital.

---

RADIUM. The London *Times* estimates the total production to date at about one ounce.

Prize Essay. The Society of Hygiene of Childhood of Paris announces a competition, closed December 31, 1913. The question to be discussed is "The Place Which Ideas of Child Culture and Hygiene Ought to Occupy in Modern Education." Manuscripts may be written in French, German, English, Italian or Spanish. They must bear a device or motto repeated on a sealed envelope containing the name and address of the competitor, and every competitor who discloses his identity will be disbarred. Essays will not be returned or even printed in their entirety and must not be published by their authors. The Society reserves the right to draw upon the best for material for a pamphlet in an educational campaign. The prizes will be awarded at the annual meeting of 1914, and will consist of medals of gold, silver gilt, silver, bronze and honorable mention. Address the President de la Societe d'Hygiene de l'Enfance, 10, rue St. Antoine, Paris 4e.

---

Medical Missionary. A physician with a college education and otherwise well qualified as a Christian gentleman, is needed at Beira, Portuguese East Africa. The successful applicant will be sent to London for a course in Tropical Medicine, then to Lisbon to learn Portuguese. The native language is said not to be difficult to acquire. A gasoline launch will be provided for missionary tours on the rivers. The climate is rather bad, so that two month's vacation will be allowed yearly at Mt. Silinda or elsewhere, and a year's furlough will be allowed every five years. Address regarding this and other missionary openings, Mr. Wilbert B. Smith, 600 Lexington avenue, New York.

---

Sterilization of the Unfit. Indiana, Washington, Colorado, Connecticut, Nevada, Iowa, New Jersey and New York have all passed laws which provide for some form of sterilization of defectives and of certain criminal types. In Pennsylvania, a similar bill was vetoed by the Governor, and in Kansas and Nebraska the experiment with such methods of dealing with sexual offenders has been temporarily abandoned. The New Jersey law has been very carefully planned to avoid abuse, but has not been in effect long enough to warrant conclusions as to its practical outcome. Indiana has given the sterilization plan a seven years' trial, and in the reformatory at Jeffersonville about three hundred men have been thus operated on.

Legislation Regarding Animal Experimentation. The press report, which was noted in the March issue, to the effect that the McClelland Anti-vivisection bill was killed in the New York Senate Judiciary Committee, by a vote of 6 to 4, was incorrect in two important particulars. First, the bill was not killed in committee but was reported favorably, April 3, by a vote of 9 to 4; secondly, it is not an anti-vivisection bill, but one whose intention is to secure a reasonable supervision of animal experimentation. A similar bill has been introduced in the Assembly by Mr. McKee. These bills have the support of the Society for the Prevention of Abuse in Animal Experimentation, President, Dr. Albert Leffingwell of Aurora, N. Y., Treasurer and Counsel, Frederick P. Bellamy, Esq., 204 Montague street, Brooklyn. This organization is in no sense one of anti-vivisectionists, although it naturally does not meet with favor from those who oppose all legislation on this subject nor from those who desire special privileges for institutional workers instead of affording equal rights and equal control to all legitimate students of physiology, pharmacology and other sciences requiring animal experimentation. It is desired that physicians interested, communicate with Mr. Bellamy. It should be clearly understood that the editor, in making these notices, and in holding the opinion that some legislative control of vivisection is inevitable and based on public sentiment and precedent along other lines, by no means advocates arbitrary restriction of scientific advance. Judgment on the merits of a bill requires not merely such reading as applies to ordinary scientific articles, but keen scrutiny, with abundant legal experience to guard against future interpretation contrary to the spirit apparently expressed.

---

Openings for Physicians. If any young doctor finds that he is the victim of the inexorable law of supply and demand, we would advise him to seek a position where a genuine demand exists. Viola, Del., needs a physician. This is a town of fifty families, but there are two other villages within two miles and two more within three miles, and a thickly settled farming district with an aggregate population of 2000. Inquiries may be addressed to Mr. Henry De Blois.

Rural Grove and Sharon, N. Y., have recently lost their only physicians by death, and there are over a hundred post offices in New York where there is no physician at all. Many of these, with the surrounding country in a radius no greater than is included in an ordinary city practice, have populations several times as large as the average tributary to each physician (about 650.)

OBESITY AND HIRSUTIES. Miss Emma Groves died at Brunswick, Me., April 3, of pneumonia, aged 46. She was five feet six tall, five feet eight in waist measure, weighed 507 pounds and had a full beard.

---

ELECTRICITY CHARGES REDUCED. The State Public Service Commission, after thorough investigation, has ordered a conservative reduction in rates for Buffalo, amounting to about 25-30% for the average small consumer. The importance of this is not in the comparatively small saving for present consumers but in the anticipated extension of the use of electricity which will undoubtedly render a further reduction possible. It should be clearly understood by purveyors of public utilities that the sentiment "The greatest good to the greatest number," means, first, "A greater use by a greater number," and, secondly, "A greater profit from a cheaper service." Cheap electricity means not only more business to the community and, hence, greater prosperity, but it means cleanliness, saving of time, better health by eliminating carbon dioxid due to the older method of producing heat and light by oxidation, and with due care in distributing currents, fewer fires. It is only one or two moves from cheap electricity to a lower death rate.

---

THE NATIONAL HIGHWAYS COMMISSION (Charles Henry Davis, President pro tempore, S. Yarmouth, Mass.) is attempting to secure public support for a bill establishing a national commission of highways. A map has been prepared, indicating various trunk routes of travel through the U. S. It is proposed to spend about $50,000,000 in five annual instalments, to assist the states in developing a system of good roads aggregating 50,000 miles. Private contributions are also solicited in sums of from fifty cents or a dollar a year from subscribing associate members up to a single payment of $25,000 for the founder. It is estimated that there are about 25,000,000 farm horses and mules, 1,600,000 horse-drawn vehicles and 850,000 automobiles in the United States. The cost of construction of roads is estimated at five to twenty thousand dollars a mile (brick). The ultimate economy of the expense is shown. While we approve of the plan, with due regard for economy and proper distribution of the expense, we may say that the argument as to the diminished cost of supplies from the country by good roads, has not been verified in experience in Buffalo. Moreover, the general use of the automobile is not for business but for pleasure, and it has appreciably increased the difficulty of doing business in the summer by tempting business men away from their work.

Medical Expert Testimony. The Committee on Medical Expert Testimony of the New York State Medical Society has caused to be introduced into both houses of the State Legislature the following proposed bill:

"An Act to amend the judiciary law, in relation to examining physicians.

"The People of the State of New York, represented in Senate and Assembly, do enact as follows:

"Section 1. Article two of chapter thirty-five of the laws of nineteen hundred and nine (1909) entitled 'An act in relation to the administration of justice, constituting chapter thirty of the consolidated laws,' is hereby amended by adding at the end thereof a new section, to be section thirty-one, to read as follows:

"Section 31. *Examining Physicians.* In a criminal action or proceeding or in a special proceeding instituted by the state writ of habeas corpus or certiorari to inquire into the cause of detention, in which the soundness of mind of a person is in issue, the court in which or the judge or justice before whom the action or special proceeding is pending may appoint not more than three disinterested competent physicians to examine such person as to his soundness of mind at the time of the examination. Any examining physician may be sworn as a witness at the instance of any party to the action or proceeding. The compensation of such examining physician for making such examination and testifying when certified by the presiding judge or justice of the court or judge or justice making the appointment, shall be paid out of any funds available for the payment of and in the same manner as other court expenses.

"Section 2. This act shall take effect immediately."

This bill has been most carefully drawn by an expert on constitutional law at the instance of the committee. The expert who drafted the bill has taken into account the laws of all the other states and the decisions regarding them where declared unconstitutional.

It is his opinion that this bill will stand the constitutional test. He further believes that it is wiser to use it as an amendment to the judiciary law than to have it enacted as an entirely new legislation. It is sincerely hoped by the committee of the State Society that the profession of the State of New York will endorse this bill and stand back of it with a solid front. The abuses of Medical Expert Testimony have been and are, shameful, The committee deem it the better part of wisdom to confine its efforts to having the bill enacted covering only criminal cases.

The bill was introduced in the Senate on March 17th by Senator Walters of Onondaga, and in the Assembly on the same date by Assemblyman Daly.

RAISE EDUCATIONAL STANDARD. The Association of American Medical Colleges, which met in Chicago in February, adopted a resolution that hereafter students in schools belonging to the Association of American Medical Colleges must take a five-year course. There are now thirty colleges which enforce a two years' collegiate course preparatory to admittance to a medical college, and five other colleges have adopted the same rule, effective January 1, 1914. After January 1, 1914, a year of college work in physics, chemistry and a modern language will be required. Special standards for clinical work were also adopted at the meeting.

---

QUADRUPLETS. Mrs. Eisenhauer of Scarsdale, N. S., is said to have given birth to three girls weighing six pounds each and a boy weighing five and one-half pounds, early in March. Allowing for placenta and liquor amnii, this means a uterine content of 27-30 pounds. Scarsdale is not listed in Polk, so we cannot verify the report.

---

101 TUBERCULOUS CATTLE have recently been condemned by Dr. H. S. Wende, State Veterinarian for Western New York, at Springville, out of 141 examined. It would be interesting to trace the effects in the consumption of milk . (No pun intended.)

---

THE GLASCOW LISTER WARD AND MUSEUM. As a memorial to the late Lord Lister, and as a means of perpetuating his memory in a way that it is hoped will prove both interesting and instructive to every member of the medical profession for all time to come, one of the wards in the Royal Infirmary, Glasgow, in which he worked out and first put into practice the principles of Antiseptic Surgery, is to be reserved and utilized in the following way. One part of the ward is to be refurnished as it was in his time with such objects as it may be possible to acquire; while the other part is to be made into a Museum for the exhibition of anything associated with the life and work of the great master.

It is, therefore, asked that any who may have letters, pamphlets, books, or other objects of direct personal association with Lister and his work will either present or loan them to the Museum.

Professor John H. Teacher, M. D., Hon. Curator of the Museum, will be pleased to receive any objects addressed to him at the Royal Infirmary, Glasgow, Scotland.

The names of all donors or senders of objects are to be affixed to the exhibits.

Dr. A. Ernest Mayland, 12 Blythswood Square, Glasgow, is Chairman of the Museum Sub-Committee.

---

Examinations for Assistant Surgeons, Public Health Service, will be held at various places, May 5 and June 9. Candidates should address the Surgeon-General at Washington, immediately, for particulars.

